# Combination of immunotherapy and chemotherapy as first-line treatment for advanced or recurrent endometrial cancer: a meta-analysis of phase 3 trials

**DOI:** 10.1186/s12885-025-15039-2

**Published:** 2025-10-14

**Authors:** Rongxia Li, Xinmiao Zhang, Jinhai Shen

**Affiliations:** 1 Department of Gynecology, The Second Hospital of Hebei Medical University, Shijiazhuang, Hebei Province 050004 China; 2 College of Integrated Traditional Chinese and Western Medicine, Hebei University of Chinese Medicine, Shijiazhuang, Hebei Province 050091 China; 3 State Key Laboratory of Natural Medicines, China Pharmaceutical University, Nanjing, Jiangsu 211198 China; 4 Center for New Drug Safety Evaluation and Research, China Pharmaceutical University, Nanjing, Jiangsu 211198 PR China; 5 School of Basic Medicine and Clinical Pharmacy, China Pharmaceutical University, Nanjing, Jiangsu 211198 China

**Keywords:** Immunotherapy, Chemotherapy, Endometrial cancer, Phase 3 trial, Meta-analysis

## Abstract

**Background:**

The integration of immunotherapy with chemotherapy (CT) has emerged as a major focus of clinical research in recent years for patients with advanced or recurrent endometrial cancer (EC). Recent phase 3 randomized controlled trials (RCTs) have reported heterogeneous outcomes, prompting ongoing debate within the oncology community. This study aimed to evaluate the efficacy and safety of this combined approach through a meta-analysis of phase 3 RCTs.

**Methods:**

A systematic literature search identified phase 3 RCTs comparing first-line immunotherapy plus CT *versus* CT alone in advanced or recurrent EC. Meta-analyses were performed to pool hazard ratios (HRs) for progression-free survival (PFS) and overall survival (OS), odds ratios (ORs) for objective response rate (ORR), and relative risks (RRs) for adverse events (AEs).

**Results:**

Four phase 3 RCTs (*n* = 2,334) met eligibility criteria. The combination therapy significantly improved PFS (HR, 0.60; 95% CI, 0.47–0.78), OS (HR, 0.75; 95% CI, 0.65–0.87), and ORR (OR, 1.42; 95% CI, 1.17–1.73) compared to CT alone. While a modest increase in grade 3–5 AEs (RR, 1.11; 95% CI, 1.03–1.20) was observed with combination therapy, there was no significant rise in serious AEs. Subgroup analyses revealed enhanced survival benefits in patients with mismatch repair-deficient (dMMR) tumors, PD-L1-positive tumors, and recurrent disease.

**Conclusion:**

This meta-analysis demonstrates that combining immunotherapy with CT as first-line treatment for advanced or recurrent EC offers superior efficacy over CT alone while maintaining a comparable toxicity profile. Notably, greater benefits were observed in dMMR, PD-L1-positive, and recurrent disease subgroups.

**Supplementary Information:**

The online version contains supplementary material available at 10.1186/s12885-025-15039-2.

## Introduction

Endometrial cancer (EC) is a significant global health concern, ranking as the sixth most common cancer among women worldwide and the second most common gynecologic malignancy [1]. The incidence of endometrial cancer is projected to increase, driven by factors such as obesity and an aging population [2]. Current treatment strategies for advanced or recurrent EC primarily involve chemotherapy, with carboplatin and paclitaxel being the standard first-line regimen [2, 3]. However, despite these treatments, long-term outcomes remain suboptimal, with median overall survival (OS) typically less than three years [3]. The integration of immunotherapy into treatment protocols has shown promise, particularly for tumors with mismatch repair deficiency (dMMR) or microsatellite instability-high (MSI-H) status, which are more responsive to immune checkpoint inhibitors (ICI) [4–6]. This evolving therapeutic landscape underscores the need for innovative and effective treatment approaches to improve patient outcomes.

Four pivotal phase 3 clinical trials—RUBY, NRG-GY018, DUO-E, and AtTEnd—have been conducted to evaluate the efficacy of various immunochemotherapy (ICT) combinations in advanced EC. The RUBY trial assessed dostarlimab in combination with carboplatin and paclitaxel, demonstrating significant improvements in progression-free survival (PFS) and OS, particularly in the dMMR/MSI-H population [7, 8]. Furthermore, Part 2 of the RUBY trial evaluated dostarlimab plus the PARP inhibitor niraparib, which demonstrated a promising PFS benefit (hazard ratio [HR], 0.63), supporting the potential role of PARP inhibition in enhancing immunotherapy efficacy, particularly in pMMR populations [9]. The NRG-GY018 trial investigated pembrolizumab combined with carboplatin and paclitaxel, showing enhanced PFS both in dMMR and pMMR cohorts (HR 0.34 and 0.57, respectively), although the OS benefit in pMMR cases was not statistically significant (HR 0.79) [10, 11]. The DUO-E trial explored durvalumab with platinum-based chemotherapy followed by either durvalumab alone or durvalumab plus olaparib as maintenance therapy. Notably, the addition of olaparib further improved PFS (HR 0.57 vs. 0.77 with durvalumab alone), while OS results in the pMMR subgroup remain immature [12–14]. The AtTEnd trial evaluated atezolizumab combined with carboplatin and paclitaxel with maintenance until disease progression, demonstrating significant improvements in PFS overall and in the dMMR/MSI-H population, whereas no benefit was observed in the pMMR subgroup [15]. 

Although significant gains in PFS have been observed across these four studies, the OS data are not yet mature, leading to uncertainty regarding long-term benefits. Furthermore, discrepancies exist in objective response rates (ORR). Additionally, individual studies lacked the statistical power to analyze critical subgroups effectively. Therefore, a meta-analysis is necessary to systematically evaluate the efficacy and safety of ICT in the treatment of advanced or recurrent EC, and to identify the subgroups most likely to benefit from this combination therapy. This meta-analysis aims to synthesize evidence from the RUBY, NRG-GY018, DUO-E, and AtTEnd trials, to provide a comprehensive evaluation of the efficacy and safety of ICT in advanced or recurrent EC, and to quantify the magnitude of therapeutic benefit across critical subgroups. By resolving inconsistencies in trial outcomes, this study seeks to optimize clinical decision-making and inform the development of novel ICT combination strategies.

## Methods

### Protocol and reporting guidelines

The research protocol was registered on the International Prospective Register of Systematic Reviews (CRD420250652593) and adhered to the Preferred Reporting Items for Systematic Reviews and Meta-analyses 2020 checklist [16]. 

### Information sources and search strategy

A rigorously designed systematic search strategy was executed in PubMed to identify all relevant phase 3 randomized controlled trials (RCTs) published up to March 15, 2025. Search queries were developed using a combination of Medical Subject Headings (MeSH) terms and free-text keywords to optimize sensitivity and specificity. The complete search syntax is provided in Supplementary TableS1. To ensure comprehensive coverage and capture updated data from ongoing or completed trials, relevant conference proceedings were also screened for abstracts associated with the relevant studies.

### Selection criteria

For inclusion in the meta-analysis, studies had to meet the following criteria: (i) Trials had to be phase 3 RCTs comparing the combination of ICI and CT with CT alone. (ii) Participants were required to be patients with advanced or recurrent EC. (iii) Data on survival outcomes, including HRs with 95% confidence intervals (CIs), had to be available. Conversely, studies were excluded if: (i) They were not phase 3 RCTs. (ii) CT was not used as the control arm. (iii) ICI were not used in the experimental arm. (iv) They were ongoing studies without published results as of the literature review date. Only studies meeting the inclusion standards were included in the meta-analysis.

### Data collection and assessment of risk of bias

The data from all included studies were extracted and summarized by one investigator and independently verified a second reviewer. The following data were collected where possible: the name of the clinical trial, the year of its publication, the size of the study sample, the treatment protocols, the characteristics of the population, the HRs along with corresponding 95% CIs for PFS and OS, the number of patients achieving an objective response, and the incidence of grade 3–5 adverse events (AEs) and serious AEs. Moreover, details regarding the study design were gathered to evaluate the risk of bias in each study. The risk of bias was appraised thoroughly in accordance with the Cochrane bias assessment tool [17]. 

### Statistical analysis

The combined estimates were produced utilizing either a fixed-effects model or a random-effects model, contingent upon the level of heterogeneity observed. The *I²* statistic and Cochrane *Q* test were used to assess heterogeneity. Heterogeneity was considered significant if *I²* exceeded 50% and the *Q* test p-value was below 0.1. To assess therapeutic efficacy, HRs with 95% CIs for PFS and OS, and odds ratios (ORs) with 95% CIs for ORR, were calculated to obtain a pooled estimate. For the assessment of safety, relative risks (RRs) with 95% CIs for AEs were determined on a per-study basis to provide a comprehensive evaluation. To account for inadequate statistical power for homogeneity testing, subgroup analyses were conducted using random-effects model. Funnel plot and Egger’s test were used to check for publication bias. Sensitivity analyses using the leave-one-out approach were conducted to validated the robustness of the pooled results. Sensitivity analyses employing the leave-one-out method were performed to assess the robustness of the pooled results. All statistical analyses were conducted using R software (version 4.2.2), with a two-tailed p-value < 0.05 considered statistically significant.

## Results

### Study selection and characteristics of included studies

The literature search yielded 12 records. Of these, four trials satisfied the eligibility criteria and were incorporated into the analysis [7, 8, 10–15]. The PRISMA flow diagram of identifying the eligible studies is shown in Figure S1.

A total of 2,334 patients with advanced recurrent EC were included in present meta-analysis, of whom 1,249 (53.5%) received ICI plus CT and 1,085 (46.5%) CT alone. In each trial, the experimental arm received an ICI (dostarlimab in RUBY, pembrolizumab in NRG-GY018, durvalumab in DUO-E, and atezolizumab in AtTEnd) combined with carboplatin and paclitaxel, while the control arm received placebo plus carboplatin and paclitaxel. The duration of immunotherapy maintenance varied across trials: dostarlimab was administered for up to 3 years in the RUBY trial, pembrolizumab for up to 14 cycles in NRG-GY018, while both durvalumab in DUO-E and atezolizumab in AtTEnd were continued until disease progression or unacceptable toxicity. The characteristics of each trial are listed in Table 1.

**Table 1:** Main characteristics of the included clinical trials

Study	Year	Design	Intention-to-treat population	Regimens		Population characteristics	mPFS (Mon) HR for PFS (95% CI)	mOS (Mon) HR for OS (95% CI)
				Exp arm (IO maintenance)	Ctrl arm			
RUBY [7, 8] (NCT03981796)	2023 2024	Phase 3, double-blind, placebo-controlled RCT (randomization 1: 1)	494 Exp: 245 Ctrl: 249	Dostarlimab plus carboplatin and paclitaxel (up to 3 years)	Placebo plus carboplatin and paclitaxel	Patients with primary advanced stage III or IV or first recurrent endometrial cancer. 51.4% ≥ 65 years, 77% White, 60.2% ECOG 0. dMMR/MSI-H tumors accounted for 23.9%, while pMMR/MSS tumors represented 76.1%.	NR vs. 7.7 0.64 (0.51–0.80)	44.6 vs. 28.2 0.69 (0.54–0.89)
NRG-GY018 [10, 11] (NCT03914612)	2023 2025	Phase 3, double-blind, placebo-controlled RCT (randomization 1: 1)	pMMR Cohort: 588 Exp: 294 Ctrl: 294	Pembrolizumab plus carboplatin and paclitaxel (up to 14 cycles)	Placebo plus carboplatin and paclitaxel	Patients with advanced or recurrent endometrial cancer (stage III/IVA, IVB, or recurrent). Median age 66 years (dMMR) and 65.5 years (pMMR), 64.3% (dMMR) and 67% (pMMR) had ECOG 0. dMMR tumors accounted for 27.6%, while pMMR tumors represented 72.4%.	13.1 vs. 8.7 0.57 (0.44–0.74)	27.96 vs. 27.37 0.79 (0.53–1.17)
			dMMR Cohort: 222 Exp: 110 Ctrl: 112				NR vs. 8.3 0.34 (0.22–0.53)	NR vs. NR 0.55 (0.25–1.19)
DUO-E [12–14] (NCT04269200)	2023 2024	Phase 3, double-blind, placebo-controlled RCT (randomization 1: 1)	479 Exp: 238 Ctrl: 241	Durvalumab plus carboplatin and paclitaxel (until disease progression or intolerance)	Placebo plus carboplatin and paclitaxel	Patients with advanced/recurrent endometrial cancer. Durvalumab Arm: Around 48% ≥ 65 years, 65% ECOG 0. 20% had dMMR/MSI-H tumors, 80% pMMR/MSS, and about 71% were PD-L1 positive. Control Arm: Around 48% ≥ 65 years, 70% ECOG 0. 20% had dMMR/MSI-H tumors, 80% pMMR/MSS, and 63% were PD-L1 positive.	10.2 vs. 9.6 0.71 (0.57–0.89)	NR vs. 25.9 0.77 (0.56–1.07)
AtTEnd [15] (NCT03603184)	2024	Phase 3, double-blind, placebo-controlled RCT (randomization 2: 1)	551 Exp: 362 Ctrl: 189	Atezolizumab plus carboplatin and paclitaxel (until disease progression or intolerance)	Placebo plus carboplatin and paclitaxel	Patients with advanced/recurrent endometrial cancer (median age 67 years). 23% dMMR/MSI-H, 74% pMMR/MSS; 47% PD-L1 positive; 68% ECOG 0.	10.1 vs. 8.9 0.74 (0.61–0.91)	38.7 vs. 30.2 0.82 (0.63–1.07)

*Abbreviation*: *mPFS* median progression-free survival, *mOS* median overall survival, *HR* Hazard ratio, *CI* Confidence interval, *RCT* Randomized clinical trial, *Exp* experimental arm, *Ctrl* control arm, *IO* immunotherapy, *ECOG* Eastern Cooperative Oncology Group, *dMMR/MSI-H* mismatch repair-deficient/microsatellite instability-high, *pMMR/MSS* mismatch repair-proficient/microsatellite stable, P*D-L1* programmed death-ligand 1

### Efficacy in intention-to-treat (ITT) population

**Fig. 1:**
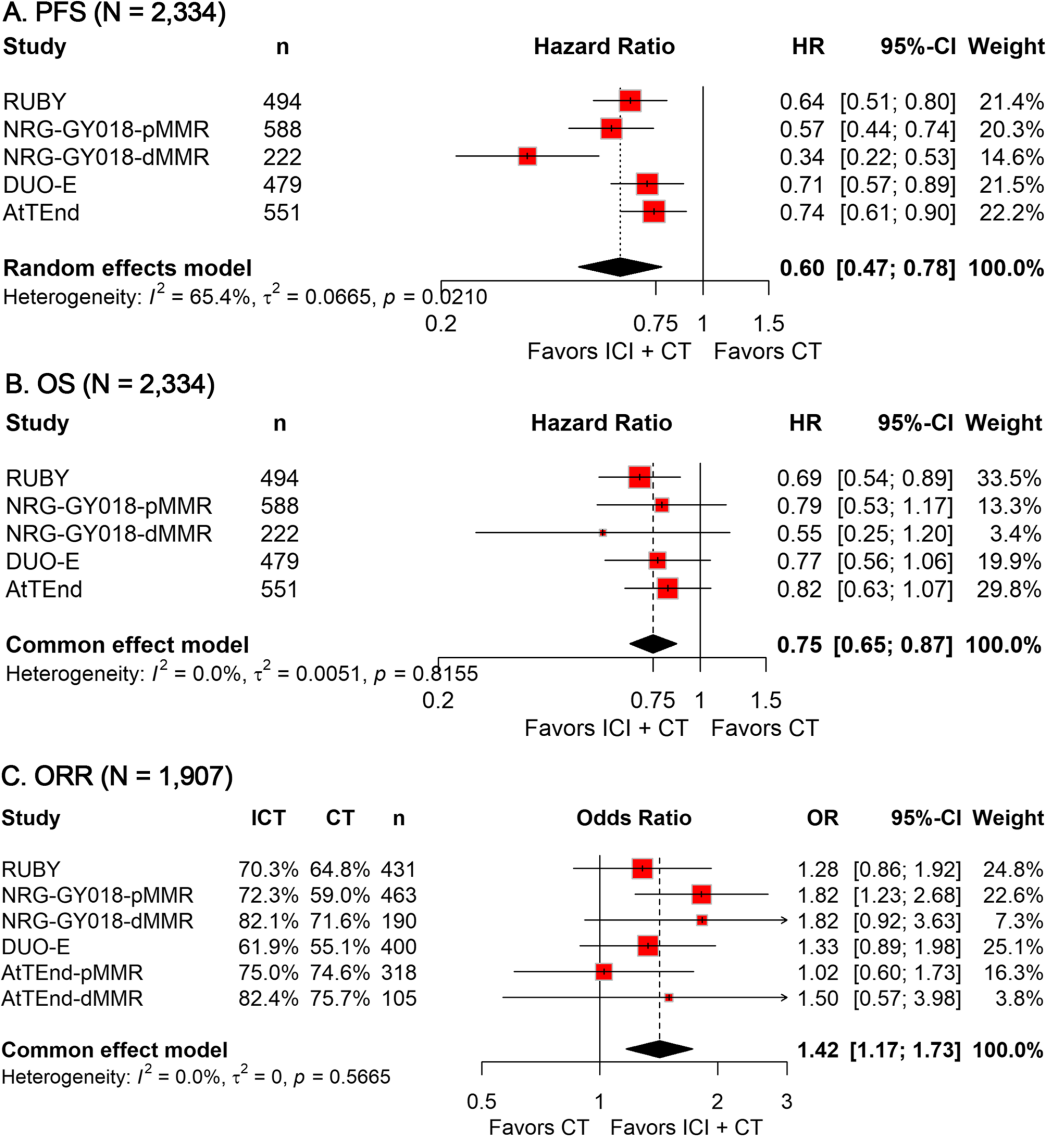
Forest plots showing the pooled results of PFS (**A**), OS (**B**), and ORR (**C**) in the ITT population when comparing ICI plus CT with CT alone

Data on PFS, OS, and ORR were available from all four trials, encompassing a total of 2,334 patients. The meta-analysis demonstrated consistent therapeutic advantages of ICI plus CT combination regimens over CT monotherapy across all endpoints. Pooled analyses showed a statistically significant 40% reduction in disease progression risk with ICT combinations (HR, 0.60; 95% CI, 0.47–0.78; Fig. 1A), alongside a 25% lower mortality risk (HR, 0.75; 95% CI, 0.65–0.87; Fig. 1B). Furthermore, the addition of ICI to CT was associated with a 1.42-fold increased probability of achieving objective tumor responses (OR, 1.42; 95% CI, 1.17–1.73; Fig. 1C), confirming the superior antitumor activity of combination therapy.

### Safety in ITT population

**Fig. 2:**
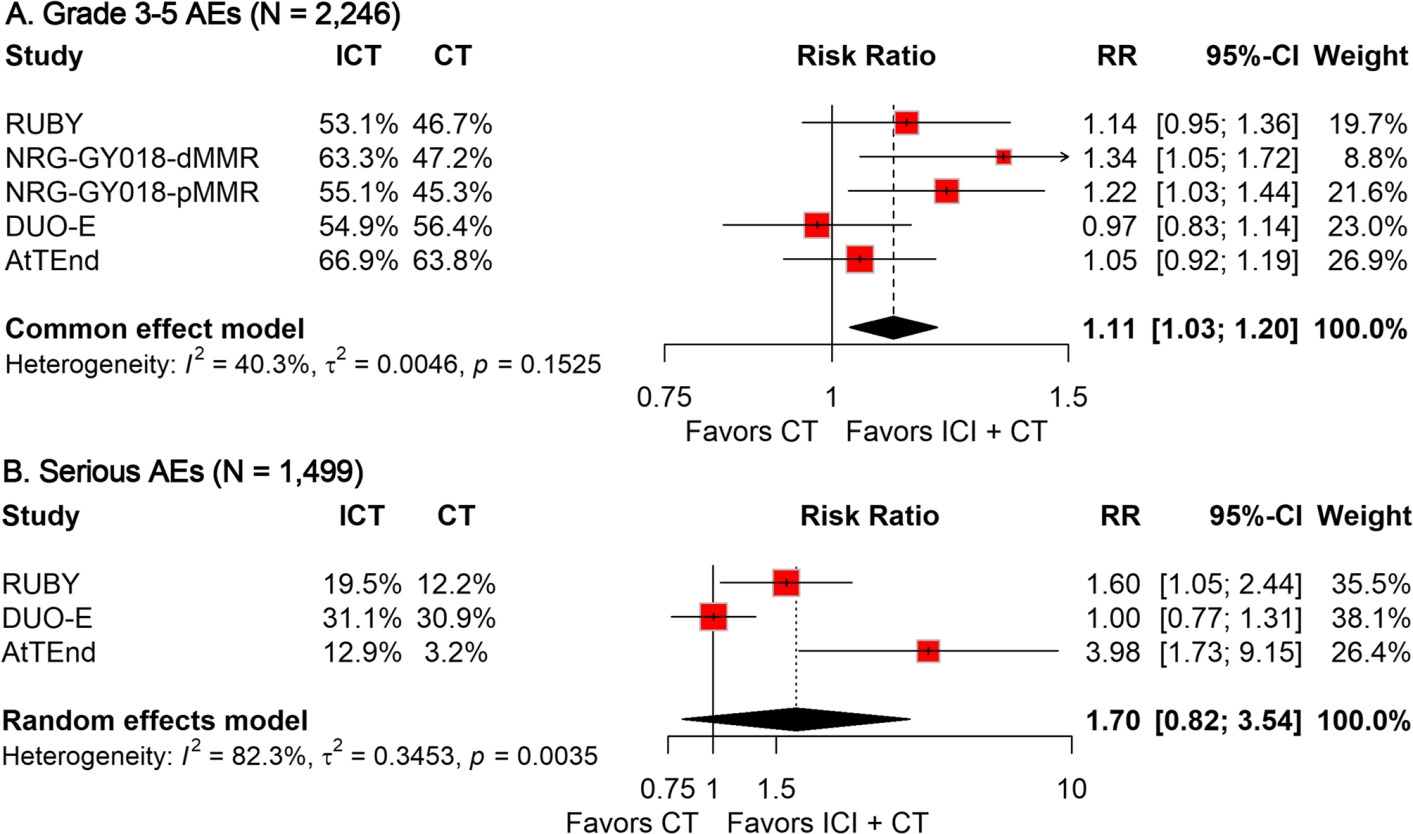
Forest plots depicting the pooled results of grade 3–5 AEs (**A**) and serious AEs (**B**) in the ITT population during the comparison of ICI plus CT with CT alone

In the group of 1,217 patients receiving ICI combined with CT, 716 individuals (58.8%) suffered from grade 3–5 AEs, compared to 540 of the 1,047 patients (51.6%) treated only with CT. The pooled RR revealed that the addition of ICI to CT significantly elevated the risk of grade 3–5 AEs (RR, 1.11; 95% CI, 1.03–1.20; Fig. 2A). Safety data regarding serious AEs of ICI plus CT *versus* CT alone were available in three trials. The prevalence of serious AEs was 20.0% (166/832) in the ICI plus CT group and 16.3% (109/667) in the CT-alone group. The pooled analysis results indicated that patients receiving the combination treatment did not experience a significantly increased risk of serious AEs (RR, 1.70; 95%CI, 0.82–3.54; Fig. 2B).

### Subgroup analysis

Because individual studies lacked sufficient power to analyze key subgroups, we performed subgroup analyses stratified by mismatch repair (MMR) status, PD-L1 expression, and disease status to clarify the efficacy of ICI combined with CT in specific populations and to inform precision treatment strategies.

**Fig. 3:**
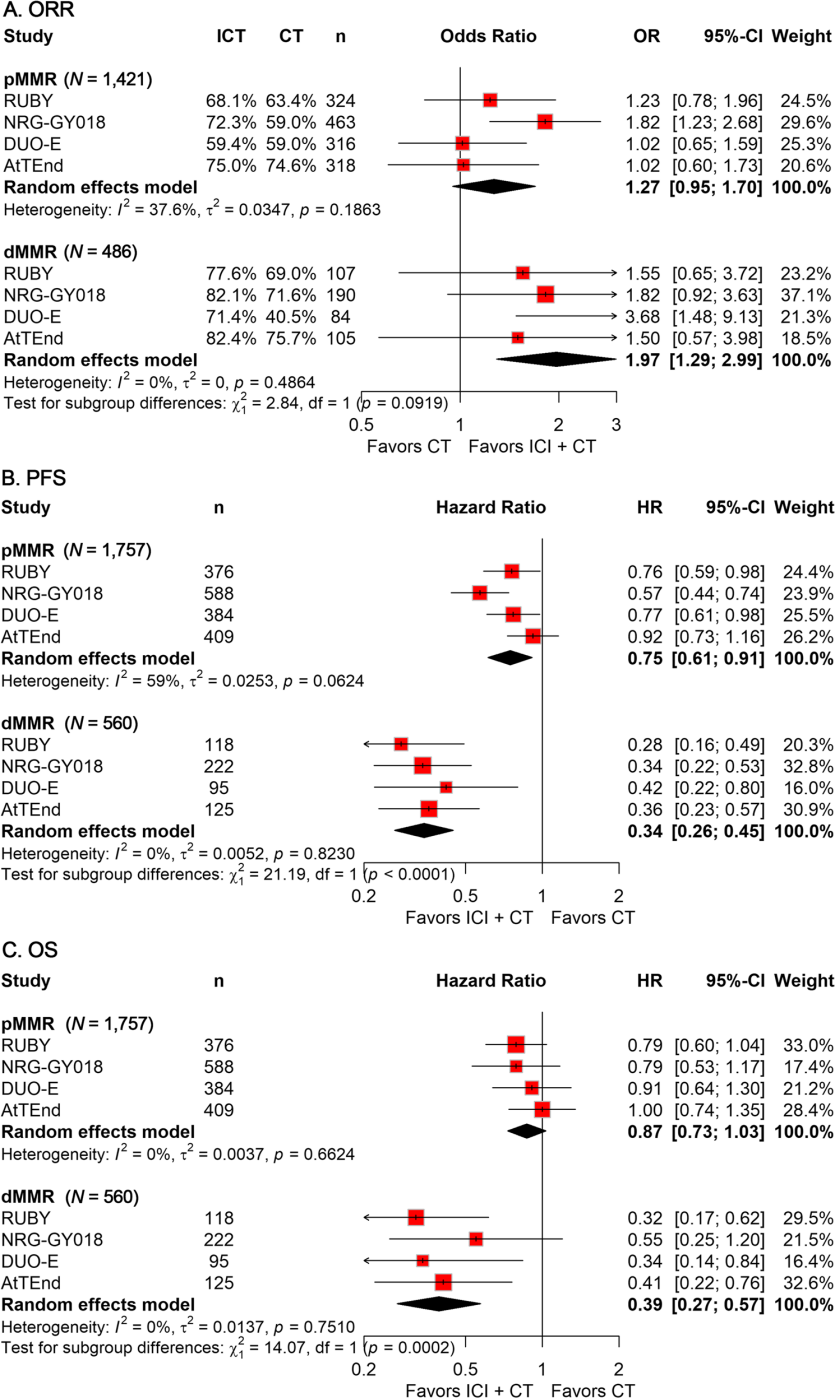
Forest plots displaying the results of ORR (**A**), PFS (**B**), and OS (**C**) of subgroup analyses stratified by MMR status

Subgroup analyses stratified by MMR status revealed that adding ICI to CT significantly improved ORR (OR, 1.97; 95% CI, 1.29–2.99; Fig. 3A), PFS (HR, 0.34; 95% CI, 0.26–0.45; Fig. 3B), and OS (HR, 0.39; 95% CI, 0.27–0.57; Fig. 3C) in patients with dMMR. Conversely, in patients with proficient MMR (pMMR), only PFS was significantly prolonged (HR, 0.75, 95% CI: 0.61–0.91; Fig. 3A), with no significant differences observed in ORR or OS.

**Fig. 4:**
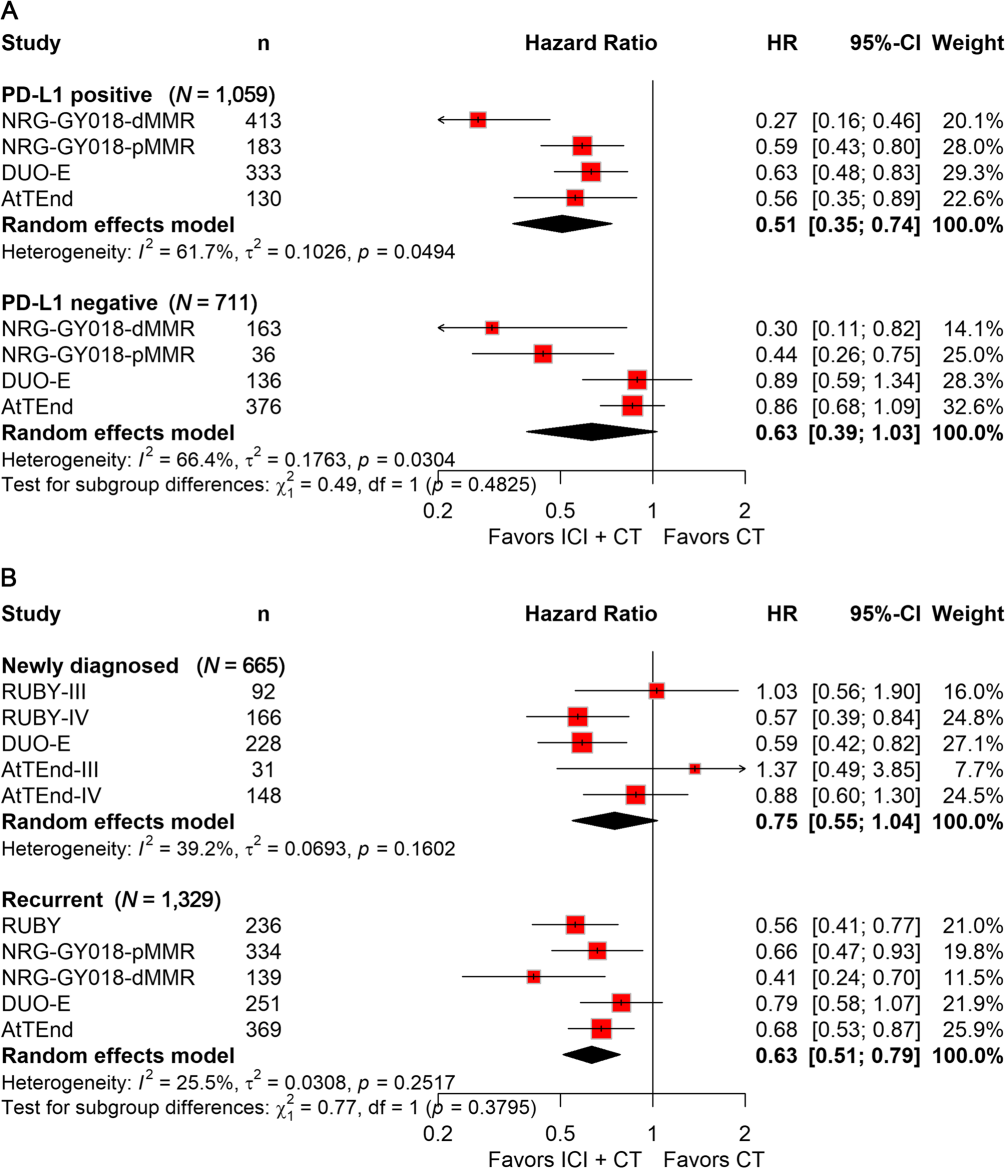
Forest plots displaying the results of PFS of subgroup analyses stratified by PD-L1 expression (**A**) and disease status (**B**)

In PD-L1-positive tumor patients, the ICI plus CT combination significantly prolonged PFS (HR, 0.51; 95% CI, 0.35–0.74; Fig. 4A). By contrast, no significant PFS difference was detected in PD-L1-negative tumor patients (Fig. 4A). While we analyzed PFS in PD-L1–positive and –negative subgroups, ORR data stratified by PD-L1 were not consistently reported across the four trials. Specifically, RUBY and NRG-GY018 only reported ORR by MMR status, not PD-L1; DUO-E reported PFS by PD-L1 but did not provide ORR for these subgroups; and AtTEnd noted that PD-L1 positivity was associated with longer PFS but likewise did not present ORR data by PD-L1. Given this lack of harmonized ORR reporting, a pooled ORR analysis by PD-L1 status was not feasible. This limitation underscores the need for future studies to consistently report efficacy endpoints, including ORR, across biomarker-defined subgroups.

Subgroup analyses stratified by disease status showed that ICI addition to CT significantly improved PFS (HR, 0.63; 95% CI, 0.51–0.79; Fig. 4B) in recurrent disease patients, whereas no significant differences were observed in newly diagnosed patients (HR, 0.75; 95% CI, 0.55–1.04; Fig. 4B).

To statistically assess the differential treatment effect across subgroups, we performed tests for interaction. A significant interaction was observed between treatment effect and MMR status for both PFS (*p*-interaction < 0.0001) and OS (*p*-interaction = 0.0002), confirming that the benefit of adding immunotherapy to CT was significantly greater in the dMMR population compared to the pMMR population. In contrast, the tests for interaction between treatment effect and both PD-L1 status (positive vs. negative; *p*-interaction = 0.4825) and disease status (newly diagnosed vs. recurrent; *p*-interaction = 0.3795) did not reach statistical significance.

### Assessment of risk of bias and sensitivity analysis

The risks of bias in the included trials were visually presented in Figure S2. All included trials exhibited low risk of bias across critical domains (selection, performance, detection, attrition, reporting), attributable to their rigorous design features (prospectively registered protocols, double-blinded intervention allocation, and endpoint assessment by independent committees). Additionally, the funnel plot and Egger’s test indicated no evidence of significant publication bias (Figure S3). Sensitivity analyses verified the stability of the combined outcomes. (Figure S4).

## Discussion

This meta-analysis of four phase 3 RCTs provides compelling evidence that combining immunotherapy with CT significantly improves survival outcomes in patients with advanced or recurrent EC, establishing this approach as a transformative advancement in first-line treatment. The integration of ICI with platinum-based CT demonstrated a 40% reduction in disease progression risk (HR, 0.60) and a 25% mortality risk reduction (HR, 0.75) compared to CT alone, alongside a clinically meaningful improvement in ORR. These findings align with the growing body of evidence supporting ICT combinations in solid tumors, particularly those with biomarker-defined vulnerabilities, such as dMMR or PD-L1 positivity [18–20]. Importantly, the safety profile of ICT remained manageable, with no significant increase in serious AEs, underscoring its feasibility as a frontline regimen.

The enhanced efficacy observed in dMMR/MSI-H and PD-L1-positive subgroups underscores the critical role of tumor biology in guiding therapeutic decisions. dMMR tumors, characterized by hypermutability and neoantigen burden, create an immunogenic microenvironment that amplifies the activity of ICI [6]. Our pooled analysis revealed striking benefits in this population, with a 66% reduction in progression risk (HR, 0.34) and a 61% mortality risk reduction (HR, 0.39), consistent with prior studies demonstrating the superiority of ICI in dMMR/MSI-H EC [21, 22]. These results reinforce the importance of universal MMR testing in EC to identify patients most likely to derive long-term benefit from ICT. For PD-L1-positive tumors, the observed PFS improvement (HR, 0.51) aligns with the mechanistic rationale of PD-1/PD-L1 axis blockade. However, due to the immaturity of OS data across the included trials, the meta-analysis of OS in this subgroup was not performed.

In contrast, the modest PFS benefit in pMMR tumors (HR, 0.75) without ORR or OS improvements highlights the limitations of current ICI in immunologically “cold” EC subtypes. pMMR tumors are typically characterized by a low tumor mutational burden, limited neoantigen landscape, and sparse infiltration of effector immune cells, features that contribute to their designation as immunologically “cold” tumors [23]. This unfavorable microenvironment may explain the lack of durable benefit from ICI monotherapy or ICT in this subgroup. Frequent genomic alterations such as PTEN loss may further reinforce this immune-resistant phenotype, underscoring the need for more refined biomarkers beyond MMR and PD-L1 [24, 25]. Additionally, the lack of OS benefit in pMMR populations raises questions about the durability of ICT responses and underscores the importance of developing novel combinations—such as ICI with anti-angiogenic agents or PARP inhibitors—to convert transient clinical responses into long-term survival gains [12, 26]. Preclinical and translational studies suggest that PARP inhibition can induce DNA damage, activate the cGAS–STING pathway, and promote type I interferon signaling, thereby enhancing antigen presentation and immune cell recruitment [27, 28]. Through these mechanisms, PARP inhibitors may synergize with immunotherapy to convert pMMR tumors from “cold” to “hot,” potentially improving responsiveness. These insights provide a strong rationale for ongoing trials evaluating PARP inhibitor–ICI combinations in pMMR endometrial cancer.

Subgroup analyses revealed differential efficacy based on disease status, with recurrent EC patients deriving greater PFS benefits from ICT (HR, 0.63) compared to those with newly diagnosed advanced disease. This observation may reflect differences in tumor biology or prior treatment exposure. Recurrent EC often exhibits clonal evolution and increased genomic instability after CT, potentially enhancing immunogenicity [29, 30]. Furthermore, immunotherapy-naïve recurrent patients may represent a population more responsive to ICT than those with de novo advanced disease, where bulky tumors or immunosuppressive microenvironments could limit ICI efficacy [31, 32]. These findings suggest that ICT may be particularly advantageous in the recurrent setting, where therapeutic options are limited and prognosis is poor.

Although our pooled point estimate suggests a numerical trend towards greater PFS benefit in the recurrent disease subgroup (HR, 0.63) compared to the newly diagnosed subgroup (HR, 0.87), the test for interaction was not statistically significant (*p*-interaction = 0.3795). Similarly, the difference in effect between PD-L1 positive and negative subgroups was not statistically robust (*p*-interaction = 0.4825). The lack of formal statistical significance for these interactions may be attributed to the limited number of trials contributing to these specific analyses, which reduces the power to detect a true difference. Therefore, these particular observations, while hypothesis-generating, should be interpreted with caution.

The safety profile of ICT combinations in this meta-analysis was reassuring, with only an 11% relative increase in grade 3–5 AEs and no significant rise in serious AEs. Across the included trials, the most common grade 3–5 toxicities were hematologic events (notably neutropenia and anemia) and chemotherapy-related neuropathy, consistent with expected profiles of platinum–taxane–based regimens. These findings suggest that the addition of immunotherapy to CT does not substantially alter the overall toxicity burden compared to CT alone. This contrasts with earlier concerns that combining myelosuppressive CT with ICI might exacerbate toxicity. Nonetheless, proactive monitoring and adherence to established AE management protocols remain essential to mitigate risks and ensure treatment continuity.

The results of this meta-analysis build upon recent paradigm shifts in EC treatment. Historically, CT has been the cornerstone of advanced EC management, but its benefits are transient, with median PFS rarely exceeding 13 months [3]. The RUBY, NRG-GY018, DUO-E, and AtTEnd trials collectively validate ICT as a new standard of care, mirroring advancements seen in other malignancies [18–20]. However, critical questions remain unresolved. First, the optimal duration of ICI maintenance therapy post-chemotherapy is unclear. Indeed, the trials differed substantially in their approach to maintenance: RUBY permitted dostarlimab for up to 3 years, NRG-GY018 capped pembrolizumab at 14 cycles, whereas both DUO-E and AtTEnd continued durvalumab or atezolizumab until disease progression. These variations complicate cross-trial comparisons and may partly explain inconsistencies in OS outcomes. The extended treatment duration in RUBY raises the possibility that longer immunotherapy maintenance could translate into greater survival benefit, an issue that warrants prospective evaluation to define the optimal duration of therapy. Second, the role of dual immunotherapy (e.g., CTLA-4 + PD-1 inhibition) in EC remains unexplored in phase 3 trials, despite promising phase 2 data [33]. Finally, cost-effectiveness analyses are urgently needed to address the economic implications of widespread ICT adoption, particularly in resource-limited settings.

While this meta-analysis provides robust pooled estimates, several limitations warrant acknowledgment. First, the included trials utilized different ICIs (anti-PD-1 vs. anti-PD-L1 antibodies) and maintenance strategies, introducing clinical heterogeneity. Mechanistic differences between PD-1 and PD-L1 inhibitors could theoretically influence efficacy. Dostarlimab and pembrolizumab (PD-1 inhibitors) block both PD-L1 and PD-L2 interactions with PD-1, potentially achieving broader immune activation [34]. In contrast, PD-L1 inhibitors such as atezolizumab and durvalumab selectively block PD-L1, sparing PD-L2–mediated signaling, which may attenuate efficacy in certain tumor microenvironments. Notably, in our included trials, PD-1 inhibitors were associated with numerically greater survival benefits compared to PD-L1 inhibitors, although these observations should be interpreted with caution in the absence of head-to-head comparisons. Further translational and clinical research is warranted to delineate whether mechanistic differences between PD-1 and PD-L1 blockade translate into clinically meaningful differences in outcomes for advanced EC. Second, OS data were immature in three of the four trials, necessitating future updates to confirm survival benefits. Third, the lack of individual patient data precluded granular analyses of covariates such as prior radiation exposure, histologic subtype (e.g., carcinosarcoma), or molecular alterations. Fourth, cross-trial variations in PD-L1 assay platforms and scoring systems (e.g., combined positive score vs. tumor proportion score) complicate the interpretation of PD-L1 subgroup results. Furthermore, while our subgroup analyses are informative, the findings for the PD-L1-positive and recurrent disease subgroups are primarily derived from two trials each (DUO-E and AtTEnd for PD-L1; NRG-GY018 and AtTEnd for recurrent disease), which limits the statistical power and generalizability of these specific results. The non-significant interaction test for PD-L1 status (*p* = 0.4825) and disease status (*p* = 0.3795) underscores the preliminary nature of this finding. Additionally, the underrepresentation of racial and ethnic minorities in the included trials limits the generalizability of findings to diverse populations. Finally, none of the included trials have reported exploratory pharmacodynamic or immune biomarker analyses. The lack of such data limits our understanding of the contribution of host immunity and post-treatment immune modulation to clinical outcomes, underscoring an important avenue for future investigation.

To optimize ICT strategies in EC, several research priorities emerge. First, biomarker-driven trials should evaluate ICT in molecularly defined subgroups, such as POLE-mutated or HER2-overexpressing tumors. Second, sequential therapy approaches—for example, CT followed by ICI maintenance *versus* concurrent ICT—require comparative evaluation to balance efficacy and toxicity. Third, translational studies exploring dynamic changes in the tumor immune microenvironment during ICT could identify mechanisms of resistance and guide rational combination therapies. Fourth, patient-reported outcomes and quality-of-life metrics should be incorporated into future trials to assess the holistic impact of ICT. Finally, real-world studies are needed to validate these findings in broader populations, including elderly patients and those with comorbidities often excluded from RCTs.

## Conclusion

This meta-analysis establishes ICT as a superior first-line treatment for advanced or recurrent EC, with pronounced benefits in dMMR/MSI-H, PD-L1-positive, and recurrent disease subgroups. While the therapeutic landscape continues to evolve, these findings provide a compelling rationale for integrating biomarker testing into routine clinical practice and adopting ICT as the new standard of care for eligible patients. Ongoing research must address unresolved questions regarding optimal treatment duration, biomarker refinement, and strategies to extend benefits to pMMR populations.

## Supplementary Information

Supplementary Material 1.

## Data Availability

The data resulting from this study, including all generated or analyzed datasets, are contained within the published article and its supplements. For any additional data-related inquiries, the corresponding author is available to address reasonable requests upon contact.

## References

[1] Bray F, Laversanne M, Sung H, Ferlay J, Siegel RL, Soerjomataram I, et al. Global cancer statistics 2022: GLOBOCAN estimates of incidence and mortality worldwide for 36 cancers in 185 countries. CA Cancer J Clin. 2024;74(3):229–63. 38572751 10.3322/caac.21834

[2] Oaknin A, Bosse TJ, Creutzberg CL, Giornelli G, Harter P, Joly F, et al. Endometrial cancer: ESMO clinical practice guideline for diagnosis, treatment and follow-up. Ann Oncol. 2022;33(9):860–77. 35690222 10.1016/j.annonc.2022.05.009

[3] Miller DS, Filiaci VL, Mannel RS, Cohn DE, Matsumoto T, Tewari KS, et al. Carboplatin and paclitaxel for advanced endometrial cancer: final overall survival and adverse event analysis of a phase III trial (NRG oncology/GOG0209). J Clin Oncol. 2020;38(33):3841–50. 33078978 10.1200/JCO.20.01076 PMC7676887

[4] O’Malley DM, Bariani GM, Cassier PA, Marabelle A, Hansen AR, De Jesus Acosta A, et al. Pembrolizumab in patients with microsatellite Instability-High advanced endometrial cancer: results from the KEYNOTE-158 study. J Clin Oncol. 2022;40(7):752–61. 34990208 10.1200/JCO.21.01874 PMC8887941

[5] Oaknin A, Tinker AV, Gilbert L, Samouëlian V, Mathews C, Brown J, et al. Clinical activity and safety of the anti-programmed death 1 monoclonal antibody dostarlimab for patients with recurrent or advanced mismatch repair-deficient endometrial cancer: a nonrandomized phase 1 clinical trial. JAMA Oncol. 2020;6(11):1766–72. 33001143 10.1001/jamaoncol.2020.4515 PMC7530821

[6] Le DT, Uram JN, Wang H, Bartlett BR, Kemberling H, Eyring AD, et al. PD-1 blockade in tumors with mismatch-repair deficiency. N Engl J Med. 2015;372(26):2509–20. 26028255 10.1056/NEJMoa1500596 PMC4481136

[7] Mirza MR, Chase DM, Slomovitz BM, dePont Christensen R, Novák Z, Black D, et al. Dostarlimab for primary advanced or recurrent endometrial cancer. N Engl J Med. 2023;388(23):2145–58. 36972026 10.1056/NEJMoa2216334

[8] Powell MA, Bjørge L, Willmott L, Novák Z, Black D, Gilbert L, et al. Overall survival in patients with endometrial cancer treated with dostarlimab plus carboplatin-paclitaxel in the randomized ENGOT-EN6/GOG-3031/RUBY trial. Ann Oncol. 2024;35(8):728–38. 38866180 10.1016/j.annonc.2024.05.546

[9] Mirza MR, Ghamande S, Hanker LC, Black D, Raaschou-Jensen N, Gilbert L, et al. 38MO Progression-free survival (PFS) in primary advanced or recurrent endometrial cancer (pA/rEC) in the overall and mismatch repair proficient (MMR/MSS) populations and in histological and molecular subgroups: results from part 2 of the RUBY trial. ESMO Open. 2024;9(sup_5):103538.

[10] Eskander RN, Sill MW, Beffa L, Moore RG, Hope JM, Musa FB, et al. Pembrolizumab plus chemotherapy in advanced endometrial cancer. N Engl J Med. 2023;388(23):2159–70. 36972022 10.1056/NEJMoa2302312 PMC10351614

[11] Eskander RN, Sill MW, Beffa L, Moore RG, Hope JM, Musa FB, et al. Pembrolizumab plus chemotherapy in advanced or recurrent endometrial cancer: overall survival and exploratory analyses of the NRG GY018 phase 3 randomized trial. Nat Med. 2025. 10.1038/s41591-025-03566-1. 40044930 10.1038/s41591-025-03566-1 PMC12851417

[12] Westin SN, Moore K, Chon HS, Lee JY, Thomes Pepin J, Sundborg M, et al. Durvalumab plus carboplatin/paclitaxel followed by maintenance durvalumab with or without olaparib as first-line treatment for advanced endometrial cancer: the phase III DUO-E trial. J Clin Oncol. 2024;42(3):283–99. 37864337 10.1200/JCO.23.02132 PMC10824389

[13] Chon HS, Pepin JT, Sundborg M, Gold M, Kim B-G, Blank S, et al. Durvalumab plus carboplatin/paclitaxel followed by durvalumab with or without Olaparib as first-line treatment for endometrial cancer (DUO-E/GOG-3041/ENGOT-EN10): objective response rate and duration of response by mismatch repair status. Gynecol Oncol. 2024;190(supplement 1):S61–2.

[14] Baurain J-F, Chon HS, Pepin JT, Sundborg M, Gold M, Kim B-G, et al. Durvalumab plus carboplatin/paclitaxel followed by durvalumab with or without Olaparib as a firstline treatment for endometrial cancer: overall survival and additional secondary efficacy en–dpoints by mismatch repair status in the DUO-E/GOG-3041/ENGOT-EN10 trial. Gynecol Oncol. 2024;190(supplement 1):S62–3.

[15] Colombo N, Biagioli E, Harano K, Galli F, Hudson E, Antill Y, et al. Atezolizumab and chemotherapy for advanced or recurrent endometrial cancer (AtTEnd): a randomised, double-blind, placebo-controlled, phase 3 trial. Lancet Oncol. 2024;25(9):1135–46. 39102832 10.1016/S1470-2045(24)00334-6

[16] Page MJ, McKenzie JE, Bossuyt PM, Boutron I, Hoffmann TC, Mulrow CD, et al. The PRISMA 2020 statement: an updated guideline for reporting systematic reviews. BMJ. 2021;372:n71. 33782057 10.1136/bmj.n71 PMC8005924

[17] Higgins JP, Altman DG, Gøtzsche PC, Jüni P, Moher D, Oxman AD, et al. The Cochrane collaboration’s tool for assessing risk of bias in randomised trials. BMJ. 2011;343:d5928. 22008217 10.1136/bmj.d5928 PMC3196245

[18] Zhang X, Shen J, Huang M, Li R. Efficacy and safety of adding immune checkpoint inhibitors to first-line standard therapy for recurrent or advanced cervical cancer: a meta-analysis of phase 3 clinical trials. Front Immunol. 2024;15:1507977. 39712004 10.3389/fimmu.2024.1507977 PMC11659232

[19] Shen J, Ye X, Hou H, Wang Y. Efficacy and safety of immunochemotherapy in advanced triple-negative breast cancer: a meta-analysis of randomised clinical trials. Clin Oncol (R Coll Radiol). 2025;40:103783. 39955967 10.1016/j.clon.2025.103783

[20] Zhang T, Li W, Diwu D, Chen L, Chen X, Wang H. Efficacy and safety of first-line immunotherapy plus chemotherapy in treating patients with extensive-stage small cell lung cancer: a bayesian network meta-analysis. Front Immunol. 2023;14:1197044. 37435087 10.3389/fimmu.2023.1197044 PMC10331819

[21] Mamat Yusof MN, Chew KT, Hafizz A, Abd Azman SH, Ab Razak WS, Hamizan MR, et al. Efficacy and safety of PD-1/PD-L1 inhibitor as single-agent immunotherapy in endometrial cancer: a systematic review and meta-analysis. Cancers (Basel). 2023;15(16):4032. 10.3390/cancers15164032 PMC10452317 37627060

[22] Di Dio C, Bogani G, Di Donato V, Cuccu I, Muzii L, Musacchio L, et al. The role of immunotherapy in advanced and recurrent MMR deficient and proficient endometrial carcinoma. Gynecol Oncol. 2023;169:27–33. 36493574 10.1016/j.ygyno.2022.11.031

[23] Dai Y, Zhao L, Hua D, Cui L, Zhang X, Kang N, et al. Tumor immune microenvironment in endometrial cancer of different molecular subtypes: evidence from a retrospective observational study. Front Immunol. 2022;13:1035616. 36532042 10.3389/fimmu.2022.1035616 PMC9756131

[24] Vidotto T, Melo CM, Castelli E, Koti M, Dos Reis RB, Squire JA. Emerging role of PTEN loss in evasion of the immune response to tumours. Br J Cancer. 2020;122(12):1732–43. 32327707 10.1038/s41416-020-0834-6 PMC7283470

[25] Tao Y, Liang B. PTEN mutation: a potential prognostic factor associated with immune infiltration in endometrial carcinoma. Pathol Res Pract. 2020;216(6):152943. 32279917 10.1016/j.prp.2020.152943

[26] Wu X, Wang J, Wang D, Li G, Zhang J, Chen H, et al. Fruquintinib plus sintilimab in treated advanced endometrial cancer (EMC) patients (pts) with PMMR status: results from a multicenter, single-arm phase 2 study. J Clin Oncol. 2024;42(16suppl):5619.

[27] Ding L, Kim HJ, Wang Q, Kearns M, Jiang T, Ohlson CE, et al. PARP Inhibition elicits STING-Dependent antitumor immunity in Brca1-Deficient ovarian cancer. Cell Rep. 2018;25(11):2972–e805. 30540933 10.1016/j.celrep.2018.11.054 PMC6366450

[28] Kim C, Wang XD, Yu Y. PARP1 inhibitors trigger innate immunity via PARP1 trapping-induced DNA damage response. Elife. 2020;9:e60637. 10.7554/eLife.60637 PMC7486119 32844745

[29] Fuh K, Manning-Geist BL. Endometrial cancer at recurrence: to re-sequence or not to re-sequence. Gynecol Oncol Rep. 2024;53:101414. 38841264 10.1016/j.gore.2024.101414 PMC11150684

[30] Hosea R, Hillary S, Naqvi S, Wu S, Kasim V. The two sides of chromosomal instability: drivers and brakes in cancer. Signal Transduct Target Ther. 2024;9(1):75. 38553459 10.1038/s41392-024-01767-7 PMC10980778

[31] Johnson RL, Ganesan S, Thangavelu A, Theophilou G, de Jong D, Hutson R, et al. Immune checkpoint inhibitors targeting the PD-1/PD-L1 pathway in advanced, recurrent endometrial cancer: a scoping review with SWOT analysis. Cancers (Basel). 2023. 10.3390/cancers15184632. 37760602 10.3390/cancers15184632 PMC10527181

[32] Tashireva LA, Larionova IV, Ermak NA, Maltseva AA, Livanos EI, Kalinchuk AY, et al. Predicting immunotherapy efficacy in endometrial cancer: focus on the tumor microenvironment. Front Immunol. 2024;15:1523518. 39902047 10.3389/fimmu.2024.1523518 PMC11788352

[33] Lan C, Yang X, Zhao J, Zheng M, Yang F, Xie Z, et al. Cadonilimab plus lenvatinib in patients with advanced endometrial cancer: a multicenter, single-arm, phase II trial. J Clin Oncol. 2024;42(16suppl):5600.

[34] Duan J, Cui L, Zhao X, Bai H, Cai S, Wang G, et al. Use of immunotherapy with programmed cell death 1 vs programmed cell death ligand 1 inhibitors in patients with cancer: a systematic review and meta-analysis. JAMA Oncol. 2020;6(3):375–84. 31876895 10.1001/jamaoncol.2019.5367 PMC6990765

