# Supplementary material for: Combination of immunotherapy and chemotherapy as first-line treatment for advanced or recurrent endometrial cancer: a meta-analysis of phase 3 trials
Source: BMC Cancer. 2025 Oct 14;25:1579. doi: 10.1186/s12885-025-15039-2 (PMC12523153; doi:10.1186/s12885-025-15039-2)
Supplement: Supplementary file 1 — Supplementary Material 1. [file 12885_2025_15039_MOESM1_ESM.docx]

**Supplementary Material**

**Combination of immunotherapy and chemotherapy as first-line treatment for advanced or recurrent endometrial cancer: A meta-analysis of phase 3 trials**

**Table S1.** Details of searching strategy.

**Figure S1.** PRISMA flow diagram of study selection.

**Figure S2.** Risk of bias domains and risk of bias summary.

**Figure S3.** Funnel plot and Egger’s test for objective response rate.

**Figure S4.** Sensitivity analyses for included studies on progression-free survival, overall survival, and objective response rate examined by leaving-one-out approach.

**Table S1.** Details of searching strategy.

| **Database** | **Search strategy** |
| --- | --- |
| ***PubMed*** | (("Immune Checkpoint Inhibitors"[MeSH Terms] OR ("immunotherapy"[MeSH Terms] OR "immunotherapy"[All Fields] OR "immunotherapies"[All Fields] OR "immunotherapy s"[All Fields]) OR ("ctla 4 antigen"[MeSH Terms] OR ("ctla 4"[All Fields] AND "antigen"[All Fields]) OR "ctla 4 antigen"[All Fields] OR "ctla 4"[All Fields]) OR "PD-1"[All Fields] OR "PD-L1"[All Fields] OR ("ipilimumab"[MeSH Terms] OR "ipilimumab"[All Fields]) OR ("tremelimumab"[Supplementary Concept] OR "tremelimumab"[All Fields]) OR ("dostarlimab"[Supplementary Concept] OR "dostarlimab"[All Fields]) OR ("pembrolizumab"[Supplementary Concept] OR "pembrolizumab"[All Fields]) OR ("durvalumab"[Supplementary Concept] OR "durvalumab"[All Fields]) OR ("nivolumab"[MeSH Terms] OR "nivolumab"[All Fields] OR "nivolumab s"[All Fields]) OR ("atezolizumab"[Supplementary Concept] OR "atezolizumab"[All Fields]) OR ("avelumab"[Supplementary Concept] OR "avelumab"[All Fields]) OR ("durvalumab"[Supplementary Concept] OR "durvalumab"[All Fields]) OR ("sintilimab"[Supplementary Concept] OR "sintilimab"[All Fields]) OR ("camrelizumab"[Supplementary Concept] OR "camrelizumab"[All Fields])) AND ("chemotherapy s"[All Fields] OR "drug therapy"[MeSH Terms] OR ("drug"[All Fields] AND "therapy"[All Fields]) OR "drug therapy"[All Fields] OR "chemotherapies"[All Fields] OR "drug therapy"[MeSH Subheading] OR "chemotherapy"[All Fields] OR ("cisplatin"[MeSH Terms] OR "cisplatin"[All Fields] OR "cisplatin s"[All Fields] OR "cisplatine"[All Fields] OR "cisplatins"[All Fields]) OR ("carboplatin"[MeSH Terms] OR "carboplatin"[All Fields] OR "carboplatine"[All Fields]) OR ("paclitaxel"[MeSH Terms] OR "paclitaxel"[All Fields] OR "paclitaxel s"[All Fields] OR "paclitaxels"[All Fields]) OR ("docetaxel"[MeSH Terms] OR "docetaxel"[All Fields] OR "docetaxel s"[All Fields]) OR ("topotecan"[MeSH Terms] OR "topotecan"[All Fields]) OR ("gemcitabine"[MeSH Terms] OR "gemcitabine"[All Fields] OR "gemcitabin"[All Fields] OR "gemcitabine s"[All Fields])) AND ("Endometrial Neoplasms"[MeSH Terms] OR "Endometrial Cancer"[All Fields])) AND (clinicaltrialphaseiii[Filter]) |
| ***ASCO, ESMO, and SGO*** | ((anti-CTLA-4) OR (ipilimumab) OR (anti-PD-1) OR (anti-PDL1) OR (dostarlimab) OR (pembrolizumab) OR (nivolumab) OR (atezolizumab) OR (avelumab) OR (durvalumab) OR (tremelimumab) OR (sintilimab) OR (camrelizumab) OR ("immune checkpoint inhibitor*")) AND (chemotherapy) AND ("endometrial cancer") |

ASCO, American Society of Clinical Oncology; ESMO, European Society for Medical Oncology; SGO, Society of Gynecologic Oncology.


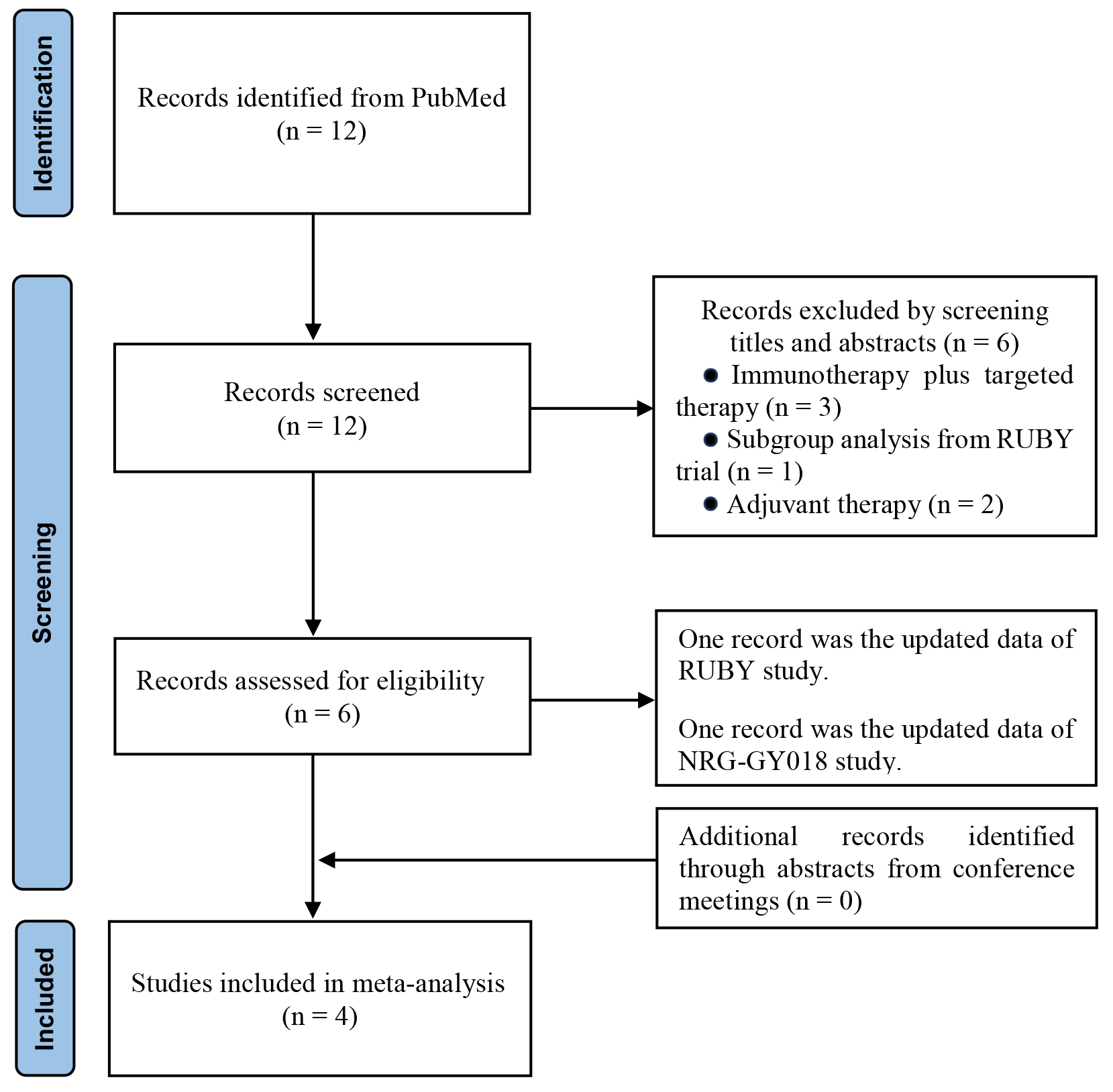


**Figure. S1.** PRISMA flow diagram of study selection.

**
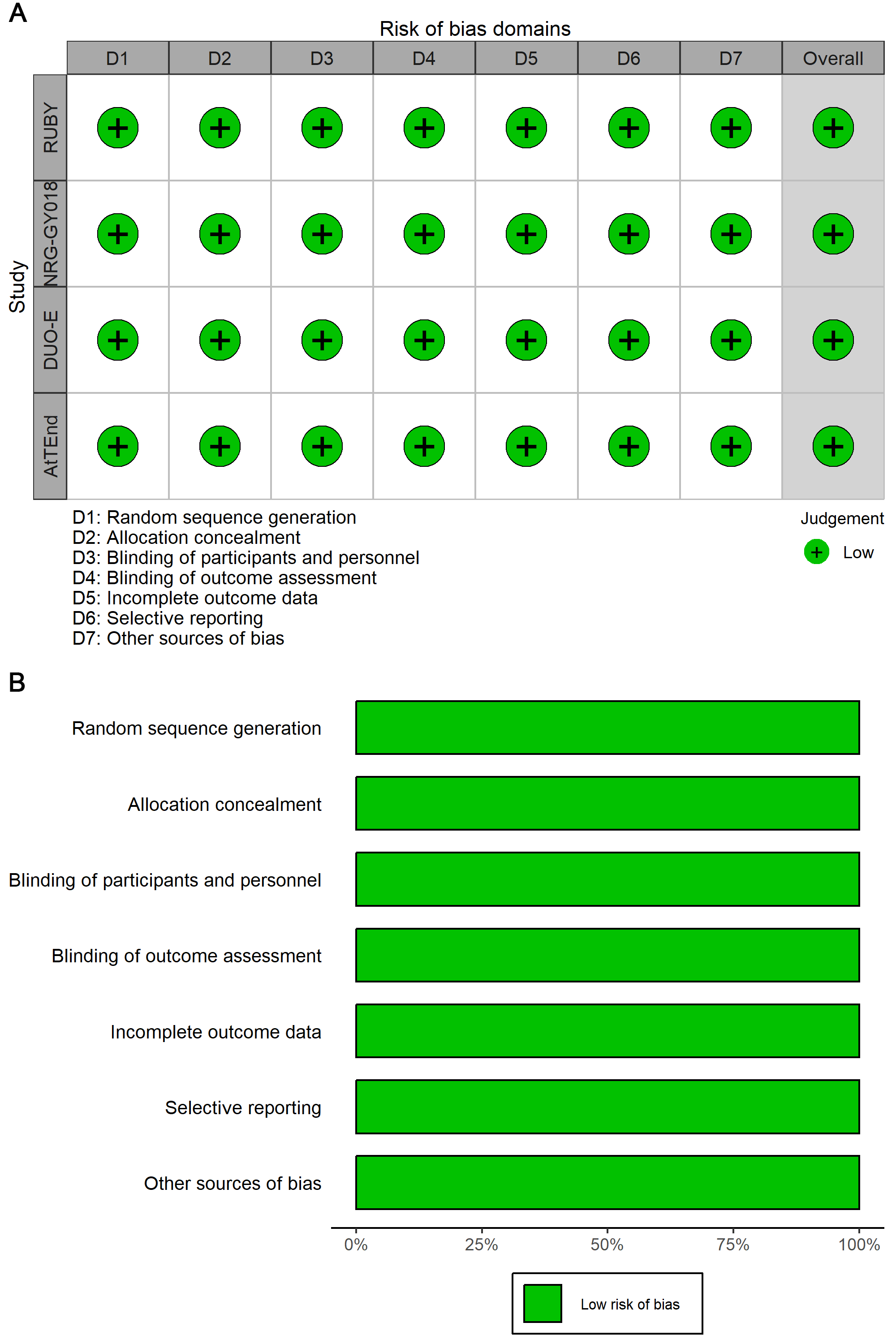
**

**Figure. S2.** Risk of bias domains and risk of bias summary.


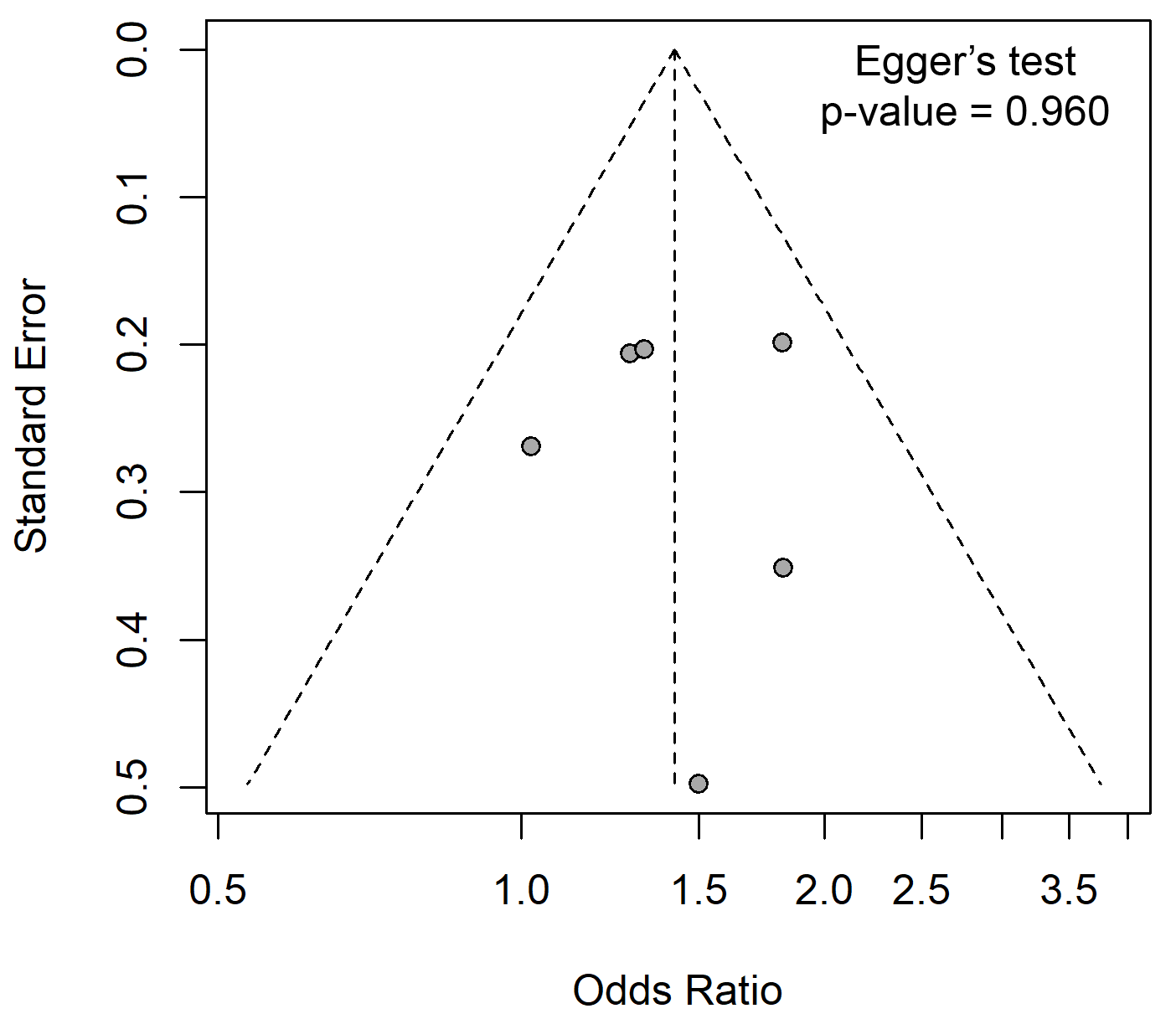


**Figure S3.** Funnel plot and Egger’s test for objective response rate.


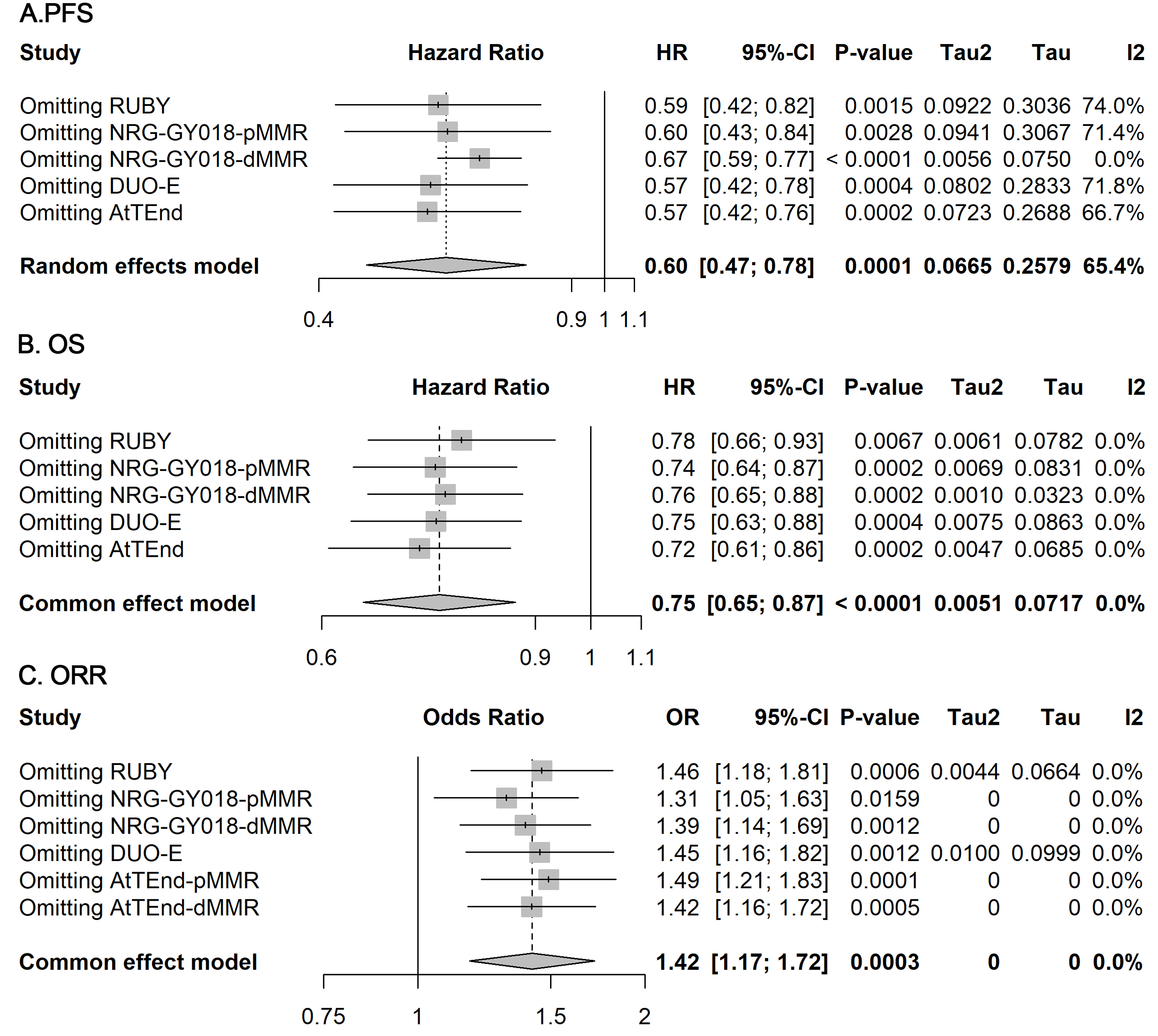


**Figure S4.** Sensitivity analyses for included studies on progression-free survival, overall survival, and objective response rate examined by leaving-one-out approach.
